# A Numerical Analysis Model for the Interpretation of In Vivo Platelet Consumption Data

**DOI:** 10.1371/journal.pone.0055087

**Published:** 2013-01-28

**Authors:** Ted S. Strom

**Affiliations:** 1 Department of Pathology and Laboratory Medicine, Memphis Veterans Administration Medical Center, Memphis, Tennessee, United States of America; 2 Department of Pathology and Laboratory Medicine, University of Tennessee Health Sciences Center, Memphis, Tennessee, United States of America; Institut National de la Santé et de la Recherche Médicale, France

## Abstract

Unlike anemias, most thrombocytopenias cannot be separated into those due to impaired production and those due to accelerated consumption. While rapid clearance of labeled platelets from the bloodstream can be followed in thrombocytopenic individuals, no model exists for quantitatively inferring from autologous or allogeneic platelet consumption data what changes in random consumption, lifespan dependent consumption, and platelet production rate may have caused the thrombocytopenia. Here we describe a numerical analysis model which resolves these issues. The model applies three parameter values (a random consumption rate constant, a lognormally-distributed platelet lifespan, and the standard deviation of the latter) to a matrix comprising a series of platelet cohorts which are sequentially produced and fractionally consumed in a series of time intervals. The cohort platelet counts achieved after equilibration of production and consumption both enumerate the population age distribution and sum to the population platelet count. Continued platelet consumption after production is halted then serves to model in vivo platelet consumption data, with consumption rate in the first such interval defining the equilibrium platelet production rate. We use a least squares fitting procedure to find parameter values which best fit observed platelet consumption data obtained in WT and thrombocytopenic WASP(-) mice. Equilibrium platelet age distributions are then ‘grafted’ into the matrix to allow modeling of the consumption of WT platelets in WASP(-) recipients, and vice versa. The optimal parameter values obtained indicate that random WT platelet consumption accounts for a larger fraction of platelet turnover than was previously suspected. Platelet WASP deficiency accelerates random consumption, and a trans effect of recipient WASP deficiency contributes to this. Application of the model to clinical data will allow distinctions to be made between thrombocytopenias due primarily to impaired platelet production and those due to acceleration of random or lifespan-dependent platelet consumption.

## Introduction

Accelerated platelet consumption rates are thought to underlie multiple types of thrombocytopenia, including immune thrombocytopenic purpura (ITP), thrombotic thrombocytopenic purpura (TTP), heparin induced thrombocytopenia (HIT), and disseminated intravascular coagulation (DIC). No current diagnostic test, however, is capable of directly distinguishing thrombocytopenias due to rapid platelet clearance from those due to impaired platelet production. A major stumbling block in this area is the lack of a mathematical model capable of simultaneously quantifying the effect of changes in random platelet consumption processes, and lifespan-dependent platelet consumption, from net *in vivo* platelet consumption data. Such a model would also allow inference of the platelet population turnover rate (i.e. the platelet production rate), and would allow the interpretation of data obtained with allogeneic as well as autologous platelets.

*Random* platelet consumption occurs in association with hemostasis, but can also occur due to uptake by splenic macrophages, hepatic macrophages (Kupffer cells), or hepatocytes [1]. *Lifespan dependent* platelet consumption is mediated by a platelet intrinsic process that terminates in apoptosis [2]. Efforts to quantify the sum of these two types of process typically involve *ex vivo* labeling of platelets with a radioisotope (such as ^111^Indium) or a fluorescent marker (such as CMFDA), injecting them into a recipient, and following the rate at which they are cleared from the circulation. Alternatively, platelets can be labeled *in vivo* by injection of sulfo-NHS-biotin, and their clearance can again be followed over time. For both types of study, the resultant curves would be linear if age-dependent clearance predominated, or exponential if random clearance predominated. They are a hybrid of the two in most circumstances [3], [4], [5], [6], [7], and quantifying the contributions of the two types of process is problematic.

The Mills-Dornhorst equation [8]–[9],originally intended to model red cell survival, has been used to interpret in vivo platelet consumption data. It is:
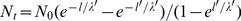
(1)Applied to platelet consumption, the terms of the equation can be described as follows: N_0_ is the number of platelets in a circulating population at time zero; N_t_ is the number of those platelets remaining in circulation at time t; l is the mean platelet lifespan in circulation, equivalent to the population lifespan or 1/(TR), where TR is the population turnover rate; l’ is the intrinsic lifespan of the platelet; λ’ is the lifespan which would result from random consumption processes if the intrinsic lifespan were infinite. 1/λ’ is the rate of random platelet consumption.

Although this equation incorporates the two types of kinetic mechanism just described, it has several significant drawbacks. First, its derivation assumes that platelets are consumed instantly when they reach their lifespan. The equation consequently generates in vivo consumption curves which acutely intersect the baseline. Actual consumption data typically demonstrates an asymptotic approach to the baseline, as might be expected if the lifespans of individual platelets are distributed about a mean, and if the spontaneous apoptosis and clearance of senescent platelets occurs at an accelerated but random rate.

Second, the equation does not allow an explicit solution for the random consumption rate constant (1/λ’). Instead, λ’ is recognized as an implicit function in the following expression [9]:
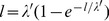
(2)A third drawback of the Mills-Dornhorst equation is its inapplicability to platelet consumption studies in which the donors and recipients demonstrate different platelet counts (i.e. most clinical platelet transfusions). This is due to its failure to account for what Dornhorst described as “delayed effects of the first environment as well as effects of the second.” [9] The most obvious of these “delayed effects” is the different age distributions of the donor and recipient platelets in such studies, which would alter the absolute rate of age-dependent platelet consumption in recipients. Thus it is unclear how to interpret studies of the consumption of platelets from thrombocytopenic individuals in normal recipients, although many such studies have been reported [10] [3] [11] [12], [13].

Similarly, Dornhorst’s concern about effects of the second (recipient) environment was reinforced by the finding that random platelet consumption becomes a more dominant component of net platelet turnover in thrombocytopenic individuals [3]. This means that any study demonstrating more rapid consumption of normal platelets in a thrombocytopenic recipient (which we have observed in the case of WT platelets in WASP(-) mice [14]) would not demonstrate that increased platelet consumption *causes* the thrombocytopenia, because a similar result would be expected as a *consequence* of the thrombocytopenia. Because the fraction of nascent platelets is expected to increase in either circumstance, interpretation of measurements of “immature” platelets is similarly problematic (this measurement increases in either context [15]).

A number of compromise efforts have been made to get around the limitations that Dornhorst recognized. For example, λ’ can be ‘mapped’, in relation to the other variables in the above equations, under circumstances in which those variables are measurable or thought to be constant. Taking this approach, Hanson and Slichter (1985) [3] and Tomer (1991) [4] used platelet consumption rate measurements in patients with bone marrow failure to estimate random (1/λ’) and lifespan-dependent (l’) platelet consumption rates in normal and ITP patients, respectively. Part of their compromise, however, was the use of a second model (the “multiple hit” model) to estimate platelet population lifespan (l). The assumptions underlying the multiple hit model are not compatible with those underlying the Mills-Dornhorst equation - and they do not appear to apply to platelet turnover.

Specifically, the multiple hit model [16] assumes that platelet consumption occurs as a consequence of a sequence of random interactions of platelets with one or more undefined environmental factors. This is currently the most widely used model for the interpretation of clinical data [7], [17], [18], [19], [20], [21], [22]. Unlike random and lifespan-dependent consumption, this model is not supported by any well characterized physical process, and a model based solely on lifespan-dependent platelet consumption demonstrates a better fit to in vivo platelet consumption data [5]. The latter model was specifically designed, however, for data obtained via in vivo labeling of platelets (via two sequential labeling methods), and cannot be applied to data obtained with ex vivo labeled platelets. More importantly, it makes no effort to take into account random platelet consumption processes. Others have relied on empirically allocating exponential and linear consumption rate constant values to platelet consumption data based on how well either type of equation fits the data (the “weighted mean” method) [23]. It is not known, however, whether this descriptive approach reflects the actual results that such simultaneous processes would generate in vivo.

A simple exponential (random) consumption model is also sometimes used when the platelet consumption curve appears (by eye) to be purely exponential [24], [25], [26], an approach recommended in the 1970’s by an international panel [27]. There are however no agreed upon criteria for deciding when platelet consumption is sufficiently non-exponential to invalidate this method.

These limitations are a major stumbling block in reaching the most basic pathophysiologic conclusions about most thrombocytopenias: whether they are due primarily to impaired production or accelerated consumption. Here we describe a numerical analysis model of platelet consumption which resolves this problem.

## Materials and Methods

### Reagents

CMFDA (“celltracker green”) and BMQC (“celltracker violet”) were obtained from Invitrogen. PE-anti-mouse CD41 was obtained from BD Biosciences. PGE-1 was obtained from Sigma.

### Mouse strains

WASP(-) mice originally derived by Snapper et al. [*28*] were crossed onto the C57Bl/6J background for at least 8 generations. All donors and recipients in this study were males. Platelet and reticulated platelet counts were routinely performed on WASP(-) mice prior to their use as platelet donors or recipients. All mice were bred and maintained in specific pathogen free environments at the Memphis VA Medical Center.

### In vivo platelet consumption assays

Platelet preparation, labeling of platelets with CMFDA, injection of labeled platelets, and quantification of the fraction of peripheral blood platelets labeled was performed as previously described [29]. In some cases, platelets labeled with BMQC (“celltracker violet”, Invitrogen) were co-injected with CMFDA-labeled platelets. Briefly, platelets were prepared (from blood obtained via tail clipping) using ficoll step gradients. Platelets at 2.9×10^5^ per ul were fluorescently labeled in modified tyrode’s buffer (20 mM Hepes, 137 mM NaCl, 13.8 mM NaHCO_3_, 2.5 mM KCl, 0.36 mM NaH_2_PO_4_-H_2_O, 5.5 mM glucose, 0.25% bovine serum albumin, 1 mM MgCl_2_) supplemented with 1 ug/ml PGE-1, and 1.25 uM CMFDA or 1.25 uM BMQC at 37 degrees C for 20 minutes. After addition of five volumes modified tyrode’s buffer +1 ug/ml PGE-1, platelets were centrifuged at 2000 RCF for 10 minutes at room temperature, resuspended in modified tyrode’s buffer, and counted using a Beckman-Coulter (Fullerton, CA, USA) Model Z2 particle count and size analyzer. Labeled platelets or mixtures of labeled platelets were brought to 2.5×10^5^ per ul and injected via tail vein into the recipients described in the text. Typical injection volumes of 400 to 600 ul yielded platelet dosages of 1 to 1.5×10^8^ platelets, resulting in most cases in labeling of 2% to 4% of circulating platelets at T_0_ (5 minutes). Recipients were bled retro-orbitally at T_0_ and at the intervals shown in the figures. In some cases the fraction of platelets labeled was determined after gating platelets via forward vs. side scatter. In others, gating also employed the addition of a PE-CD41 marker. All flow cytometric data was analyzed using Flowjo software (Tree Star Inc., Ashland, OR). All animal studies were approved by the institutional animal care and use committee of the Memphis VA Medical Center (protocol #281).

### Consumption of Indium-111 -labeled platelets

Platelets prepared as described above were resuspended at approximately 3×10^5^ per ul and incubated with 200 uCi ^111^In-oxyquinoline (GE Healthcare) per 1×10^9^ platelets at 37^0^ for 30 minutes, centrifuged at 6,000 g for 5 minutes at room temperature, and resuspended in modified tyrode’s buffer. A mock-labeling reaction was used to estimate platelet recovery from this step via Coulter counting. Specific activities of the labeled platelets ranged from 3×10^7^ to 4×10^7^ cpm per platelet. Approximately 1.2×10^8^ platelets in a net volume of 500 ul were injected per recipient. Total cpm per ul of whole blood was measured with a gamma counter at 5 minutes post injection. CPM were measured again, and corrected for decay, at the times shown in the figures.

### Computational methods

All calculations were performed using Microsoft Excel on standard iMAC desktop computers. Recurrent calculations were written into Excel “macros” using visual basic programming language. Three dimensional plots of LML values were performed with Qtiplot (http://soft.proindependent.com/qtiplot.html).

## Results

### Model

The numerical analysis model is constructed on the assumption that two kinetically distinct processes result in platelet consumption: a random destruction process (RD), designed to model the sum of hemostatic as well as other random platelet consumption processes, and applicable to all circulating platelets; and a platelet lifespan (LS). We assume that lifespan-dependent platelet consumption is determined by a series of internal pro-apoptotic processes as well as (random) recognition of apoptotic platelets by receptors on phagocytes. For this reason, LS is assumed to be lognormally distributed, an assumption supported by empirical cell fate observations in similar systems [30], [31]. It is then necessary to introduce a third parameter (SD, the standard deviation of ln(LS)) to model such a distribution. We will show (below) (1) that the rate of platelet population consumption can be modeled numerically for any set of values of RD (%/hr), LS (hr), and SD; (2) that the difference between such a model curve and observed consumption data can be quantified as the sum of squared residuals (SS); and (3) that by generating SS values for a large range of possible combinations of RD, LS, and SD values (in a hypothetical volume termed “parameter space”, or PS), and identifying a minimum value in the resultant array of SS values, we can identify the parameter values which optimally describe in vivo platelet consumption data.

**Figure 1:**
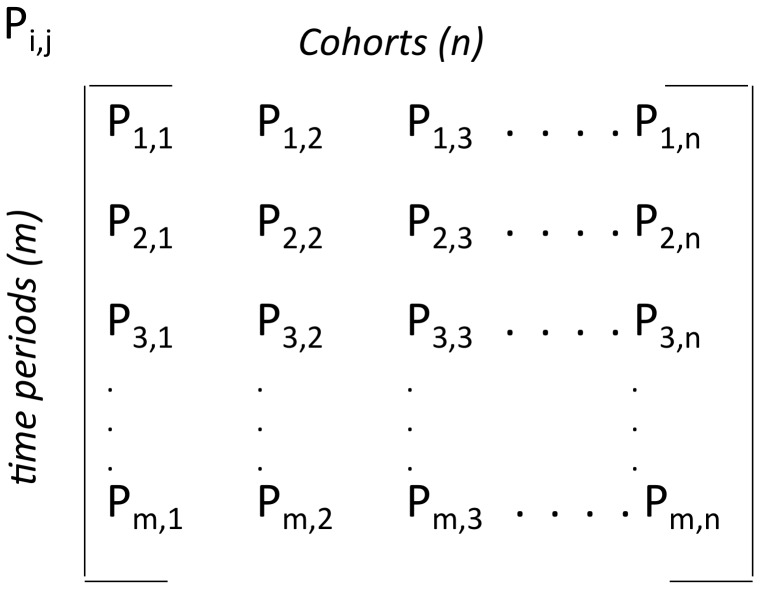
Design of platelet population matrix.

To perform that modeling, we constructed (in a spreadsheet) a dynamic population of platelet cohorts (produced hourly at a user defined production rate PR), each of which is consumed (hourly) by the aforementioned processes. This is shown schematically (**figure 1**) as a two dimensional matrix of dimensions n and m, in which the columns (1 to n) represent the concentrations (K/ul) of platelets in cohorts which are produced at time (i = j), and consumed at hourly intervals (j = i+1, j = i+2, …j = m) by two types of process (random and lifespan-dependent). The entry P_i,j_ denotes the concentration of platelets in cohort (j) at time (i).

Random consumption at the end of a given interval (RD(i)) is equal to RD (%/hr)xP(i). The cumulative amount of platelets in a cohort consumed by random consumption processes is tracked in a separate column as cumulative random destruction (CRD(i)).

Lifespan dependent consumption at the end of each interval is calculated using the excel probability density function *lognorm.dist*, applied to those platelets in the cohort not consumed by random processes ( = PR-CRD_i_). The variables used by *lognorm.dist* are LS, SD, and the age of the cohort (i–j). The resultant value is the lifespan dependent consumption amount (LSDC(i)).

Thus at interval (i+1), P = P(i) - RD(i+1) - LSDC(i+1). This value is calculated for each cell in the matrix. Range limitations are placed on the calculations to prevent the generation of negative P values.

**Figure 2:**
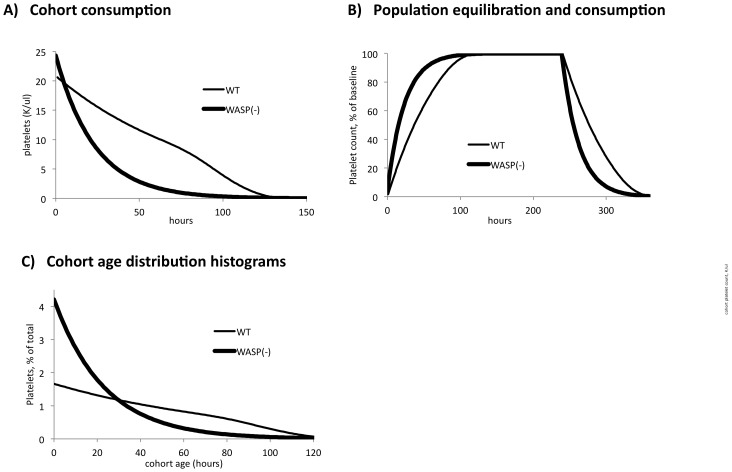
Model cohort and population platelet kinetics. A) The two model platelet cohorts shown were generated at 20.6 K/ul/hr (WT) and 24.3 K/ul/hr (WASP(-)), and consumed hourly at the following parameter values: WT, RD = 1.16%/hr, SD = 1.20, LS = 105 hr; WASP(-), RD = 4.21%/hr, SD = 0.18, LS = 150 hr. B) 240 sequential cohorts were generated hourly and consumed as in (A). Their summed hourly cohort values are shown. Note that production ceases after 240 hr. C) The platelet age distribution is taken from row the last row (time) of the equilibration phase (in this case, row i = 240).

The consumption process for individual cohorts can therefore vary from predominantly linear to exponential, as shown for two examples in f**igure 2A**. (The empirical choice of parameter values for the figure is described below). Values for sequentially produced cohorts sum to the net platelet count at time i. Over time, the net platelet count in this model increases to an equilibrium value as shown in f**igure 2B**. This value is associated with a defined platelet age distribution, as shown in **figure 2C**. Platelet production rate (PR, K/ul/hr) can be manually adjusted to generate a net platelet count consistent with observed mean values such as those we’ve reported for WT and WASP(-) mice [14].

To model in vivo platelet consumption, the matrix is extended vertically so as to contain more rows than columns, i.e. m = n+c, where a value of c = 125 hr represents a useful value in modeling murine platelet consumption. Thus at row (time) values (i>n), platelet production ceases and cohort platelet consumption continues as determined by the parameter values and the age of each cohort (j–i). Platelet population consumption from this time point continues as the sequential sums of the cohort values, generating the consumption curves seen in **figure 2B** at the end of the equilibration phase.

The validity of the model-generated consumption curves is dependent, for any set of parameter values, on the achievement of equilibrium at time j = m. We evaluate this for different sets of parameter values via an equilibration metric (e), which we define as the platelet count at time (i = n/2) divided by the platelet count at time (i = n). We assume that equilibration is functionally adequate when (e) exceeds 0.95 (i.e. the model has achieved at least 95% of its final platelet count over the first half of its equilibration phase). The validation process is described in more detail in Supporting Information (figures S1, S2, and S3).

**Figure 3:**
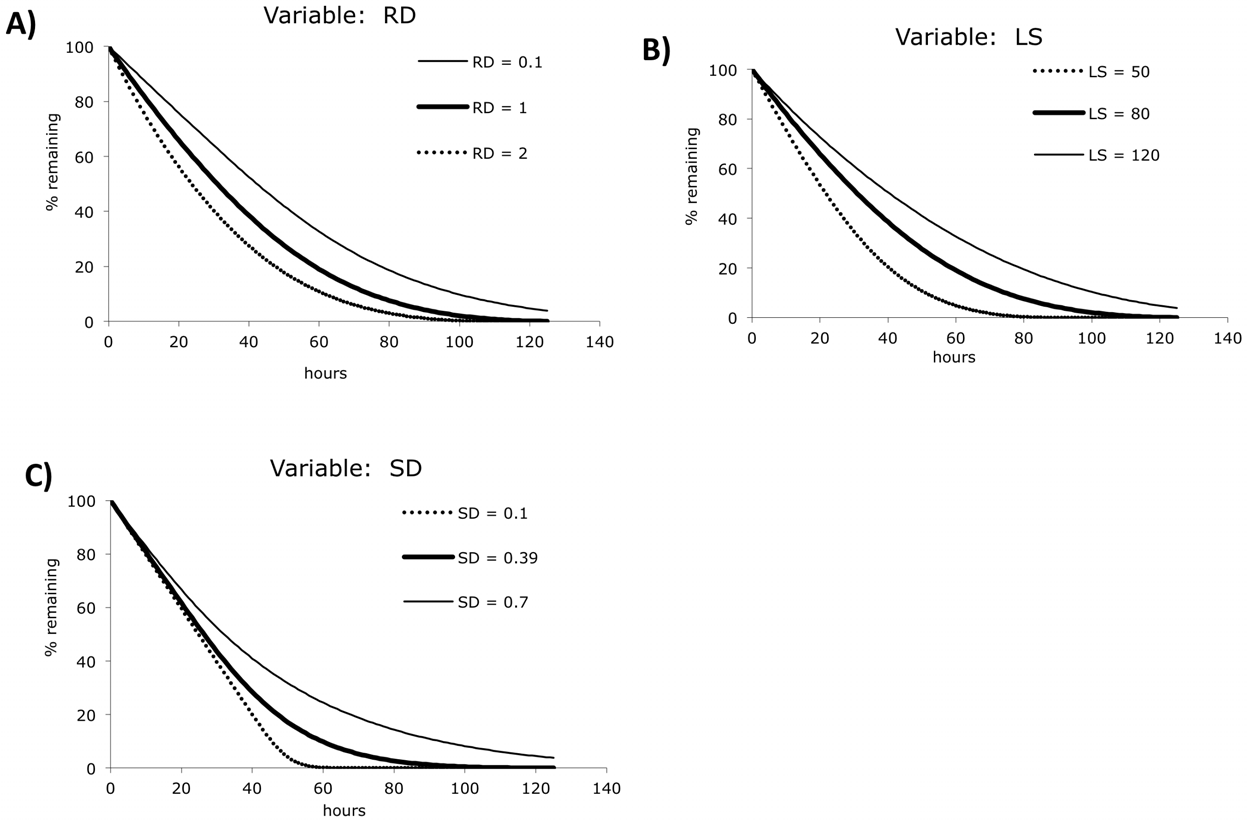
Effect of parameter value changes on the consumption curve. A 500 hour equilibration phase was used. A) Consumption at LS = 80, SD = 0.44. B) Consumption at RD = 1.0, SD = 0.44. C) Consumption at RD = 0.1, LS = 50. Equilibration metric values (e) are greater than 0.95 for all of the parameter sets shown (data not shown).

The effect of varying the three consumption parameter values on the shape of the consumption curve is shown in **figure 3**. The mid-range of these curves was chosen to roughly approximate the behavior of WT platelets in WT recipients (see below). As expected, a relative increase in RD yields a curve that begins to resemble exponential decay, while a relative decrease in LS yields more linear behavior. High SD values also cause the curve to resemble exponential decay. These results indicate that the model can emulate the not-quite-linear, not-quite-exponential platelet consumption processes seen in most published studies [3], [4], [5], [6], [7].

The model also allows direct inference of the platelet population turnover rate (hence, platelet production rate) as the rate observed in the first interval after platelet production ceases.

### Optimal parameter search method

In vivo platelet consumption data was obtained by labeling platelet preparations with a fluorescent marker (either CMFDA or BMQC); injecting them into recipient tail veins; quantifying their initial concentration (% of total platelets) in peripheral blood at 5 minutes after injection; and quantifying their concentration again at four subsequent intervals. This method, and some of the results, have been previously published [14]. The resultant data comprises a time zero (T0) measurement and four subsequent measurements of the fraction of the T0 value remaining. We have over the course of several studies obtained such data for a total of 30 WT recipients of WT platelets (WT to WT), and 18 WASP(-) recipients of WASP(-) platelets (WASP(-) to WASP(-)). These were usually obtained as control data in the course of studying other consumption rates [14], [29].

We hypothesized that a single combination of the three parameter values (RD, LS, and SD) can generate, via the model, platelet consumption curves which optimally match those observed experimentally. To determine whether the model can do this, we used a least squared residuals procedure to search for parameter values which best fit the data. The method is similar to one we previously described for a similar three-parameter search process [32]. Briefly, we evaluate 8000 points in a parameter space (PS) defined by 20 equally spaced values along three orthogonal axes. This entails generating a matrix (f**igure 1**) for each point in PS, i.e. each possible combination of parameter values. The range evaluated for each parameter is user defined. In all cases, model equilibration within the PS volume searched is confirmed (see text S1). Squared residual values are calculated for each data point (for WT to WT studies, 30×4 = 120 data points), allowing calculation of a sum of squared residuals value (SS) for all data points at each point in PS.

**Figure 4:**
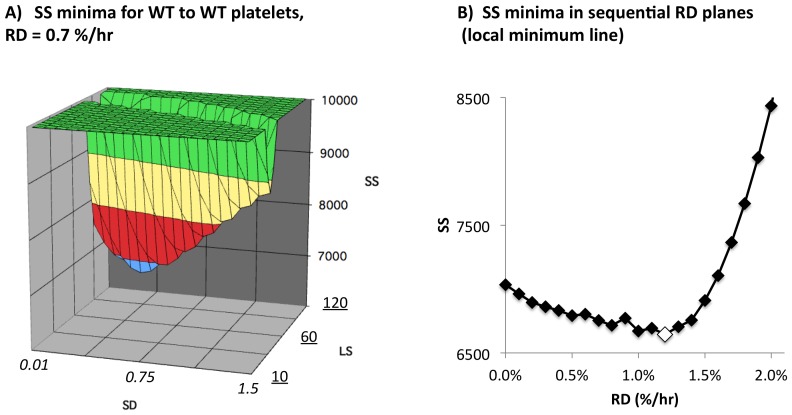
Optimal parameter search method. A) Shown are SS values for a 20×20 array of SD and LS parameter values at an RD value of 0.7%. The values shown are the squared residual values for a total of 120 WT platelet consumption measurements (30 WT recipients, four time points per recipient). The platelet consumption data used to generate the SS values is shown in figure 4. B) Shown are SS minima for a series of RD planes. The white diamond indicates the global minimum identified in this set of analyses. Details concerning the parameters space searched are in text S1.

To visualize the distribution of SS values in parameter space, we begin by evaluating all points in an RD-defined plane. The resultant SS values can be displayed as a surface in a volume defined by the axes LS, SD, and SS, as shown in **figure 4A**. We term a minimum SS value (and its associated parameter values) on such a surface a “local minimum” (LM). The LM can in turn be followed across sequential planes to define a “local minimum line” (LML) in PS. Evaluation of SS for all values on the LML is then used to identify a “global minimum” (GM) (**figure 4B**), which is subsequently resolved more precisely with a higher resolution search (see figures S4, S5, S6, S7, S8, and table S1)).

### Optimal parameters for syngeneic platelet consumption

**Figure 5:**
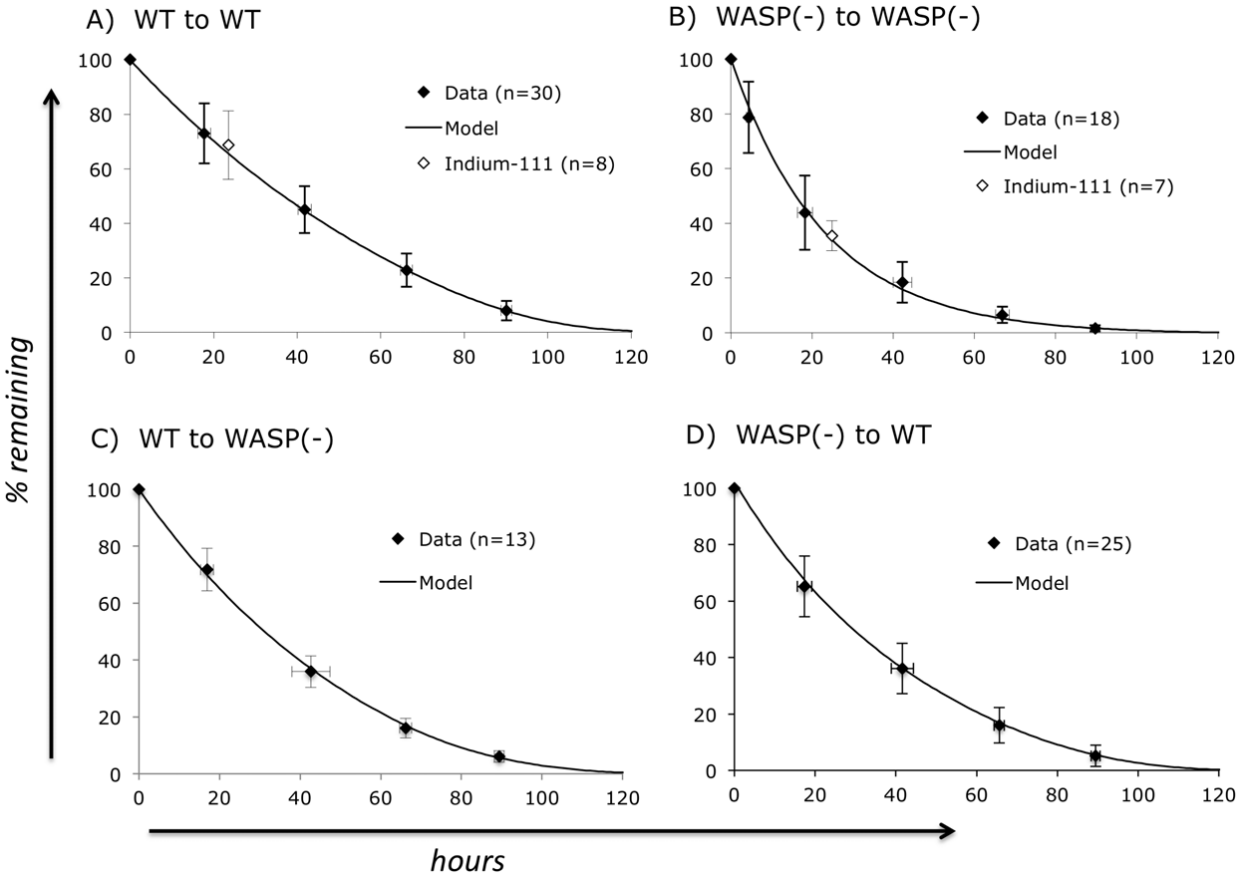
Optimal consumption curves. Black diamonds are data obtained with fluorescently labeled platelets for the “donor to recipient” data sets shown. Consumption curves are shown for the optimal parameter values shown in table 1. For allogeneic platelet consumption curves, the age distribution histograms shown in figure 2 were used to determine the optimal parameter values (see text). Each fluorescently labeled platelet data set includes “n” data points at each of the time points shown, with the exception of the WASP(-) to WASP(-) data set at 5 hours (n = 5) and at 89 hours (n = 13). Error bars are standard deviations.

Optimal parameter values (at the identified global minima) for WT to WT and WASP(-) to WASP(-) platelet consumption data are shown in **table 1**. The associated consumption curves (and the data to which they were fit) are shown in figure 5. Figure 5 also shows that similar consumption data was obtained with Indium-111 labeled platelets. This suggests that an artifactual effect of fluorescent labeling does not contribute significantly to our findings.

**Table 1:** Optimal platelet consumption parameters and associated values.

Donor	WT	WT	WASP(-)	WASP(-)
Recipient	WT	WASP(-)	WASP(-)	WT
n	30	13	18	25
RD, %/hr	1.16 (*0.09)*	**1.55** (0.03)	**4.20** (0.07)	**2.28** (0.04)
LS, hr	**105** (2.3)	105*	n/a	**106** (1.3)
SD (of ln LS)	**0.180** (0.057)	**0.275** (0.033)	n/a	**0.231** (0.043)
Platelet population turnover rate, %/hr	1.66	2.03	4.22	2.35
Random destruction, % of turnover rate	69.9	76.5	99.5	97.2
Platelet turnover rate, K/ul/hr	20.5	n/a	24.4	n/a

Values in parentheses are standard errors determined by “jacknife” resampling as described in the text. The differences between the four RD values are all significant (two sample t-test, p<0.05). The differences between columns for the SD and LS values are not significant. Population turnover rates for the allogeneic platelet consumption studies refer to the donor populations. For WT-to-WASP(-) data, LS is not greater than 105 hr (see text).

### Optimal parameters for allogeneic platelet consumption

As shown in **Figure 2C**, the model generates both an equilibrated population of platelet cohorts and a consumption curve. The latter’s shape is determined by the number or platelets (P(i)) remaining in each cohort when platelet production ceases; the associated CRD values; platelet production rate (PR, used to calculate LSDC(i)); and the parameter values applied during the consumption phase. To find optimal parameter values describing the consumption of allogeneic platelets, we “graft” the P and CRD values for each donor strain cohort (in the spreadsheet, the last row in the equilibration phase) into an identical matrix. Using the same procedures described above, and applying the PR value associated with the donor platelets, we then search for the optimal parameter values which generate a consumption curve which optimally fits the observed data. Results for consumption of WT platelets in WASP(-) recipients, and the converse type of study, are shown in **table 1** and **figure 5**. The results demonstrate both an intrinsic (cis) platelet defect induced by WASP deficiency (compare RD for WT-to-WT vs. WASP(-)-to-WT) and a recipient-dependent (trans) effect (compare RD for WASP(-)-to-WT vs WASP(-)-to-WASP(-)). The trans effect is, however, of much lower magnitude when assessed on WT donor platelets.

### Limitations on parameter estimates

To ask whether the optimal consumption curve for the WT to WT data generated by three optimal parameter values (LS, SD, RD) is significantly improved over that generated by only two parameter values (LS, SD, and RD = 0, the SS values for which are shown at the left of figure 4B), we used “jacknife” resampling [33] to calculate the standard deviation of the optimal RD value (and that of the other optimal parameter values) for each of the data sets analyzed. Details of this analysis are in supporting information (text S1, and table S2). The resultant standard deviations of the RD parameter values (table 1) clearly indicate that the RD estimates for all of the data sets analyzed are non-zero, from which we conclude that optimization of this parameter value improves significantly on modeling.

For the WASP(-) to WASP(-) data set, however, no estimate of lifespan-dependent platelet consumption could be made, as the data best fits a purely exponential (random) consumption process (see figures S5, S6, and S9). More specifically, optimal parameter values for this data set yield a consumption curve for which random consumption makes up over 99% of the net turnover rate (table 1). In contrast, lifespan-dependent consumption makes up a significant fraction of total platelet turnover for the optimal parameter set values obtained for the other data sets (table 1, and text S1).

A third limitation of the method is an inability to ask whether the recipient environment could reduce the lifespan of the donor platelets. This would entail estimating how such an effect might be distributed across the lifespan distribution of the donor platelets, while concurrently optimizing the other three parameters (discussed further in text S1). We found no evidence for any increase in the lifespan of WT platelets in WASP(-) mice (table 1).

## Discussion

The numerical analysis model we describe here allows direct inference of random and lifespan-dependent platelet consumption rates from in vivo platelet consumption data. It also allows direct inference of platelet production rate from that data. As such it solves an interpretive problem first clearly identified over 60 years ago, and to which only compromise methods have been applied thus far. No previous model of in vivo platelet consumption has been able to generate these quantitative inferences.

A recent study concluded that a lifespan-dependent platelet consumption model provided a better “fit” to in vivo murine platelet consumption data than did a model based on a hypothetical “multiple hit” mechanism [5]. Here we extend that observation by demonstrating that the combined effects of lifespan-dependent and random platelet consumption processes provide a still better fit to such normal platelet consumption data.

Because it generates an age distribution for platelets studied in syngeneic transfusion studies, the model can also be used to interpret their consumption rate in allogeneic recipients. The importance of taking the donor platelet age distribution into account is evident when the consumption of WT platelets in WASP(-) recipients (**figure 5C**) is compared to that of WASP(-) platelets in WT recipients (**figure 5D**). Although the raw data is quite similar, the optimal parameter values are markedly different (**table 1**). This is due to the strikingly different age distributions of WT and WASP(-) platelets (**figure 2**), inferred from their markedly different syngeneic consumption rates (f**igures 5A and 5B**).

Applied to a large body of in vivo platelet consumption data from 30 WT mice, the model finds that 70% of WT platelets are consumed by random processes (**table 1**). Because this differs radically from a previous estimate of 17% in humans [3], we obtained consumption data using an alternative platelet labeling method (Indium-111). This data does not differ substantially from that obtained with fluorescently labeled platelets (**figure 5**). Resampling statistics (**table 1**) generate a 95% confidence interval for the random consumption rate of 0.97 to 1.35%/hr.

These findings suggest that the rapid random platelet consumption we see is not artifactual. However, if most of this consumption resulted from a baseline requirement for normal hemostasis, we would expect that a reduction in platelet count by roughly 30% or more would result in spontaneous hemorrhages. This is not the case. Mpl(−/−) strains show platelet count reductions of over 80% [34], [35], [36], and demonstrate no abnormal bleeding. This suggests that in mice, most random platelet consumption is *not* directly involved in hemostasis.

It should be noted that a similar issue arises when human platelet consumption is considered. If 17% of human platelet turnover were required for normal hemostasis, a platelet count below that fraction of the normal human mean of approximately 290×10^9^/L (i.e. below approximately 49×10^9^/L) would be expected to result in spontaneous hemorrhage. The latter is in fact rare above a platelet count of 10×10^9^/L [37]. These considerations suggest that most random platelet consumption in humans could predominantly serve non-hemostatic biological purposes such as immune function, for which there is ample evidence [38], [39], [40].

Our results suggest that rapid random platelet consumption is the largest contributing factor to the thrombocytopenia of murine WAS, since platelet production is in fact increased (**table 1**) but is insufficient to correct their approximately 53% reduction in platelet count [14]). However, WASP(-) mice also show a nearly two-fold increase in bone marrow megakaryocytes [14] as well as a two-fold increase in spleen size that is predominantly due to extramedullary hematopoiesis [41]. This suggests that platelet production per megakaryocyte must be significantly reduced by WASP deficiency, as has been reported in *ex vivo* thrombopoiesis studies [42], [43].

The numerical analysis model also allows us to ask whether the rapid random clearance of WASP(-) platelets in WASP(-) recipients is due primarily to *cis*-acting (platelet intrinsic) or *trans*-acting factors. Our findings in **table 1** allow the following conclusions.

1. A platelet intrinsic (*cis*) defect contributes significantly to the rapid clearance of WASP(-) platelets (compare the RD of WASP(-) to WT platelets in WT recipients).
2. Platelet lifespan is not affected by WASP deficiency (compare the LS of WT vs. WASP(-) platelets in WT recipients).
3. An additional platelet extrinsic (*trans*) effect of recipient WASP deficiency results in a still more rapid random consumption rate for WASP(-) donor platelets (RD is increased by 84% for WASP(-) platelets in WASP(-) vs WT recipients).
4. The *trans* effect appears to largely require platelet WASP deficiency as well (i.e. it is a *cis/trans* effect), since it is much weaker when WT platelets are infused (RD is increased by only 34% for WT platelets in WASP(-) vs WT recipients).
5. No positive *trans* effect of the WASP(-) environment on the LS of WT platelets is evident. The current model does not allow assessment of a negative trans effect on LS. For WASP(-) platelets, we cannot assess either type of effect because random consumption in WASP(-) recipients is too rapid to allow quantification of LS.

An increased susceptibility of WASP(-) platelets to phagocytosis by splenic macrophages is the most likely mechanism for the *cis* effect. This is supported by the consistent positive impact of splenectomy on the thrombocytopenia of both clinical [44] and murine [14] WAS (in contrast to the variable efficacy of splenectomy in treating ITP), and by the increased amount of detectable platelet antigens seen in splenic macrophages in WAS patients [45]. Our observations of increased susceptibility of both murine and human WASP(-) platelets to ex vivo phagocytosis also support this mechanism [32] [46]. We do not know the molecular mechanism for either the cis or the cis/trans effect.

We note that Falet et al. [47] reported more rapid consumption of ex vivo labeled murine WASP(-) platelets in WT recipients (in terms of the fraction cleared at two hours post injection) than we report here (n = 4, vs n = 25 in our studies). Possible contributors to these different findings include the use of different platelet preparation and labeling methods, and the different genetic background (129SvEv) on which the experiments were performed. Also, their evaluation of an earlier (2 hr) time point than we did could bring the poorly understood phenomenon of reversible splenic platelet sequestration into their study.

The utility of the numerical analysis model for interpretation of murine platelet consumption data suggests that it could be applied to other types of thrombocytopenia, particularly in cases where the relative contributions of impaired platelet production and accelerated platelet consumption are not known. This is the current status of most cases of immune thrombocytopenic purpura (ITP), as the antiplatelet antibodies known to be present in many such cases can have either effect. The model should allow autologous or allogeneic platelet consumption data from such patients to be interpreted as either reflecting only an increased hemostatic burden (in the case of impaired platelet production), or that and a further increase in platelet consumption due to, for example, antibody-dependent clearance. Applied to data from individuals with bone marrow failure, it should also allow re-assessment of the fraction of human platelet consumption which occurs due to hemostasis.

## Supporting Information

Figure S1 Model equilibration metric. The behavior of the equilibration metric as a function of LS, RD, and SD is shown. (e) is defined as the total accumulated platelets at the midpoint of the equilibration phase, divided by that value at the end of the equilibration phase. Here the length of the equilibration phase was 500 hr.
(TIFF)

Figure S2 Equilibration metric contour map. (e) was evaluated for 400 points in a plane in parameter space defined by SD = 0.7. Resolution was 0.05% (RD) and 8 hr (LS). Coloration denotes points where e >0.95 (gray shading) or ≤0.95 (red). The map demonstrates that model equilibration is adequate in this SD-defined plane at the paired LS, RD values shaded in gray
(TIFF)

Figure S3 Definition of an equilibration-verified parameter space volume. See text. The contour maps (400 points per map) demonstrate that the model equilibrates adequately (over a period of 500 hours) for all RD values including zero, up to an LS value of 84 hr and an SD value of 0.7. We infer from the behavior of the contour map at a lower SD value (0.5) that equilibration is adequate (within the RD and LS limits shown in blue) for any SD value below 0.7.
(TIFF)

Figure S4 Optimal parameter search for WT to WT data. A) Three dimensional plots of the SS minima identified in 20 consecutive RD-defined planes of parameter space. B) Results shown are from searches conducted in the equilibration-verified volumes of parameter space shown in table S1. The blue line shows the SS minima as % of the “global” minimum value Triangles: PS1. Diamonds: PS2. Circles: PS3. The “global” minimum at this resolution is shown as a white diamond. The black line shows, for the same local minima, RD as % of the net turnover rate (TO). C) High resolution study of the global minimum. The equilibration-verified ranges evaluated (20 points per range) are SD 0–0.3, RD 0.96–1.35, and LS 96–115. Resolution is 5% of the range in each case. The equilibration phase for this study was 240 hr. Parameter values at the global minimum from this study are in Table 1.
(TIFF)

Figure S5 Optimal parameter search for WASP(-) to WASP(-) data. A) SS values were determined for each of 400 points in a plane in equilibration-verified parameter space defined by RD = 4.2%/hr. The LS axis range is 10 to 124 hr (resolution 6 hr)(left to right). The SD axis range is 0.01 to 4.751 (resolution 0.25). The equilibration phase duration is 500 hr. B) Three dimensional plot of the LM1 SS minima identified in 20 consecutive RD-defined planes of parameter space. The plot on the right is equivalent to that on the left, but rotated 90 degrees on the Z-axis. The LM could not be identified below an RD of 3.4%/hr. C) The same process was used to define SS values in a plane defined by RD = 1.2%/hr. The LS axis range is 10–124 hr. The SD range is 0.01 to 4.751 (resolution 0.25). LM2 in this plane is defined by SD = 1.75 and LS = 16 hr. The Equilibration phase is 500 hr. D) Three dimensional plot of the LM2 SS minima identified in 20 consecutive RD-defined planes of parameter space. The plot on the right is equivalent to that on the left, but rotated 90 degrees on the Z-axis.
(TIFF)

Figure S6 Optimal parameter search for WASP(-) to WASP(-) data. A) SS values are shown for the LML’s described in the previous figure. B) Diamonds: SS values for LML1 are shown, normalized to their minimum. Squares: RD as % of total platelet turnover is shown for the same LM points. At the global minimum identified in this study (RD = 4.2%/hr), RD accounts for over 99% of platelet turnover. C) High resolution study of LM1. The equilibration-verified parameter ranges were RD 4.12–4.13% (resolution 0.01%), LS 110–148 hr (resolution 2 hr), SD 0.01–0.3 (resolution 0.15). The global minimum identified in this study is shown in table 1.
(TIFF)

Figure S7 Optimal parameter search for WT to WASP(-) data. A) SS values were determined at the RD value shown. Ranges evaluated for SD were 0.1–0.575 (resolution 0.025; for LS, 105–124 hr left to right (resolution 1 hr). B) LM1 was evaluated in 20 consecutive RD-defined planes (range 1.1–2.1%/hr, resolution 0.05%/hr) over the LS and SD ranges described in A. All Local minima at RD values of 1.6% or less occur at an LS of 105 hr. C) Diamonds: SS values for the LML shown in (B), normalized to the global minimum. Squares: RD as % of turnover rate for the same LM points.
(TIFF)

Figure S8 Optimal parameter search WASP(-) to WT data. A) SS values were determined in the RD plane shown. Ranges evaluated for SD were 0.001–0.92 (resolution 0.03); for LS, 1–191 hr (resolution 10 hr). B) LML was evaluated in 16 consecutive RD-defined planes. Range: 0–3.8%/hr (resolution 0.2%/hr). The second 3D plot shows the LML rotated 90 degrees on the vertical axis. C) SS values for the LML shown in (B) are expressed as a percentage of their minimum value (Black diamonds). RD values for the same LML are expressed as a percentage of the total platelet turnover rate. D) A high resolution study of the global minimum in (C). Ranges evaluated are RD 1.95–2.45, LS 96–115, and SD 0.1–0.4. Resolution is 5% of each range. The resultant global minimum is shown in table 1.
(TIFF)

Figure S9 Semi-log plot of WASP(-) to WASP(-) platelet consumption data (n = 18). The Excel-generated linear regression trendline with a fixed intercept at T0 = 100% is shown. Error bars are standard deviations.
(TIFF)

Table S1 Equilibration-verified parameter space volumes (PS) searched for WT to WT data set. Resolution is 5% of the range for each axis.
(TIFF)

Table S2 PS volumes searched during jacknife resampling of data sets. Resolution is 10% of the range for each axis. For a small number of data subsets, LMs fell at the edge of the ranges shown. These were re-evaluated in adjacent volumes of PS at the same resolution.
(TIFF)

Text S1 Additional details on optimal parameter searches and other statistical methods.
(DOCX)
