# Supplementary figures and images for: A Numerical Analysis Model for the Interpretation of In Vivo Platelet Consumption Data
Source: PLoS One. 2013 Jan 28;8(1):e55087. doi: 10.1371/journal.pone.0055087 (PMC3557263; doi:10.1371/journal.pone.0055087)

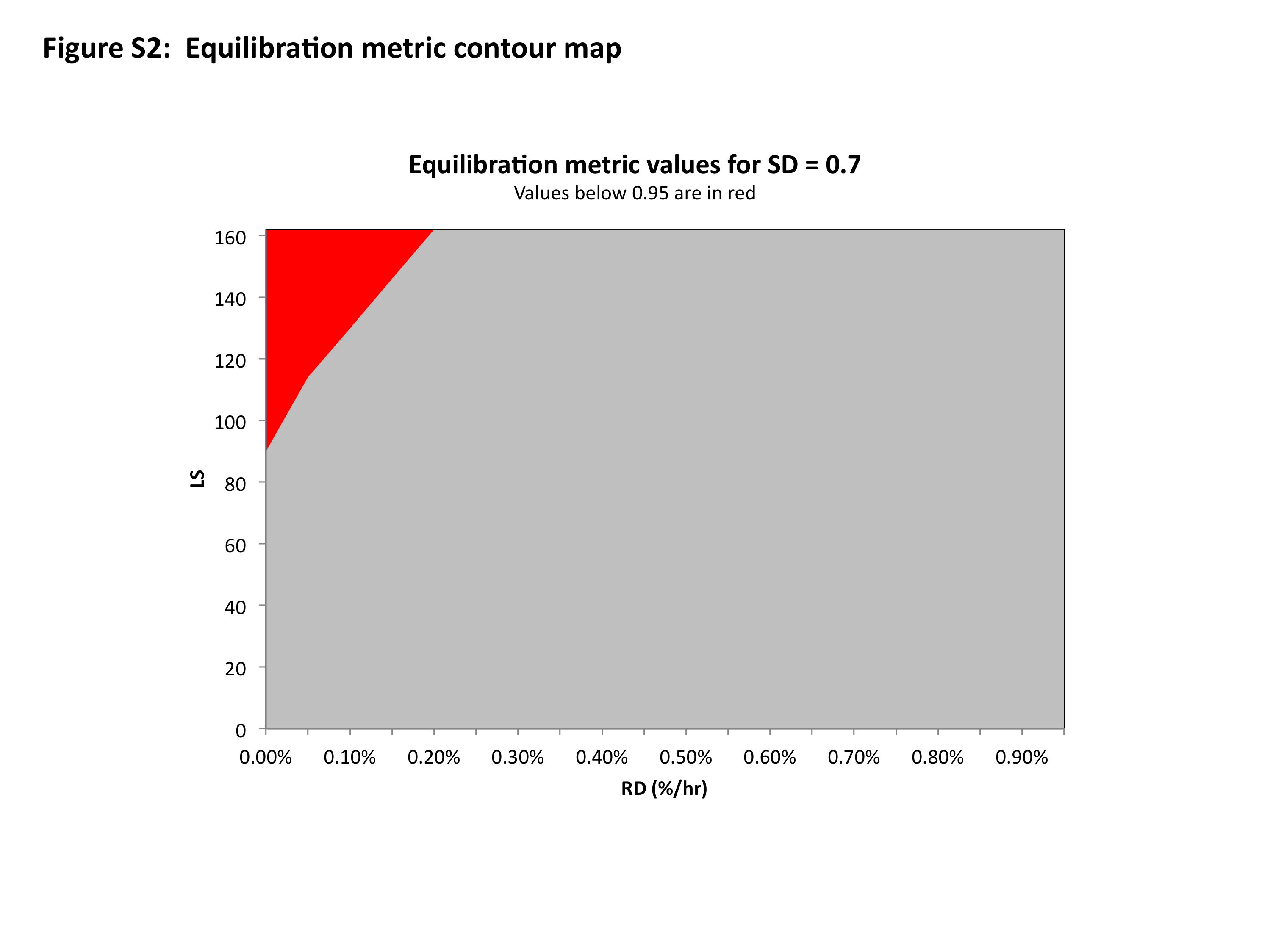

Supplement: Figure S2 — Equilibration metric contour map. (e) was evaluated for 400 points in a plane in parameter space defined by SD = 0.7. Resolution was 0.05% (RD) and 8 hr (LS). Coloration denotes points where e >0.95 (gray shading) or ≤0.95 (red). The map demonstrates that model equilibration is adequate in this SD-defined plane at the paired LS, RD values shaded in gray (TIFF) [file pone.0055087.s002.tif]

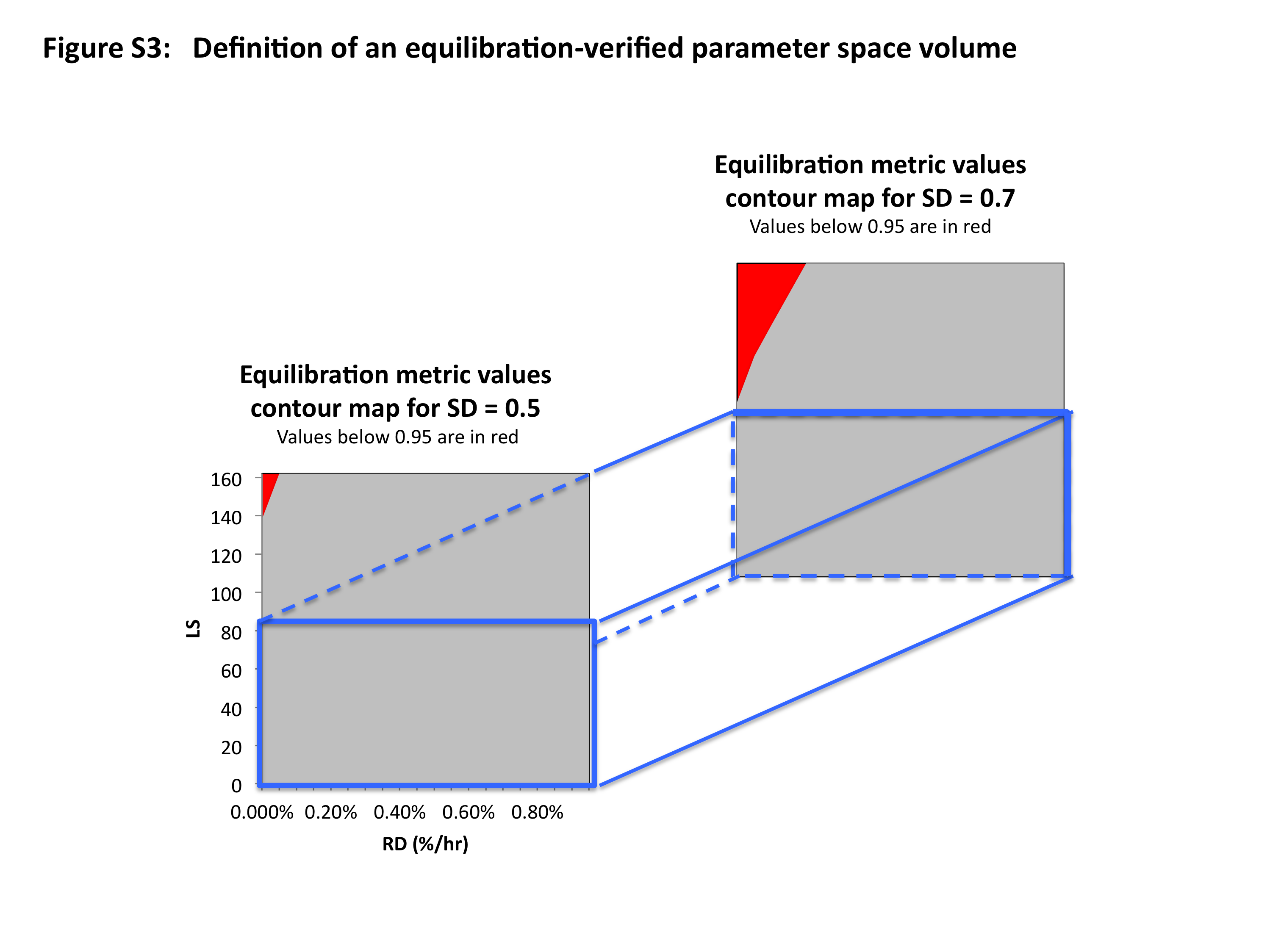

Supplement: Figure S3 — Definition of an equilibration-verified parameter space volume. See text. The contour maps (400 points per map) demonstrate that the model equilibrates adequately (over a period of 500 hours) for all RD values including zero, up to an LS value of 84 hr and an SD value of 0.7. We infer from the behavior of the contour map at a lower SD value (0.5) that equilibration is adequate (within the RD and LS limits shown in blue) for any SD value below 0.7. (TIFF) [file pone.0055087.s003.tif]

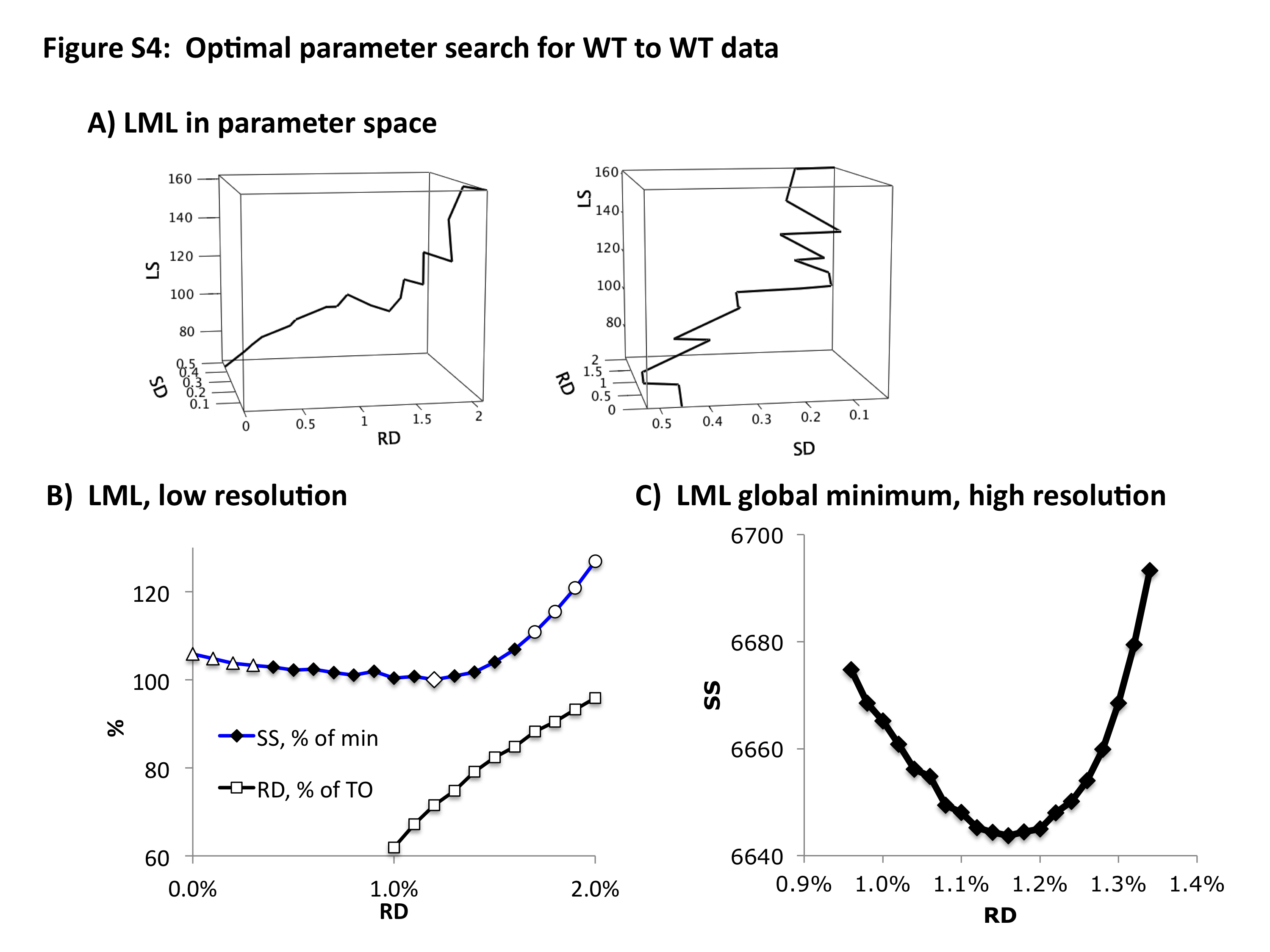

Supplement: Figure S4 — Optimal parameter search for WT to WT data. A) Three dimensional plots of the SS minima identified in 20 consecutive RD-defined planes of parameter space. B) Results shown are from searches conducted in the equilibration-verified volumes of parameter space shown in table S1. The blue line shows the SS minima as % of the “global” minimum value Triangles: PS1. Diamonds: PS2. Circles: PS3. The “global” minimum at this resolution is shown as a white diamond. The black line shows, for the same local minima, RD as % of the net turnover rate (TO). C) High resolution study of the global minimum. The equilibration-verified ranges evaluated (20 points per range) are SD 0–0.3, RD 0.96–1.35, and LS 96–115. Resolution is 5% of the range in each case. The equilibration phase for this study was 240 hr. Parameter values at the global minimum from this study are in Table 1. (TIFF) [file pone.0055087.s004.tif]

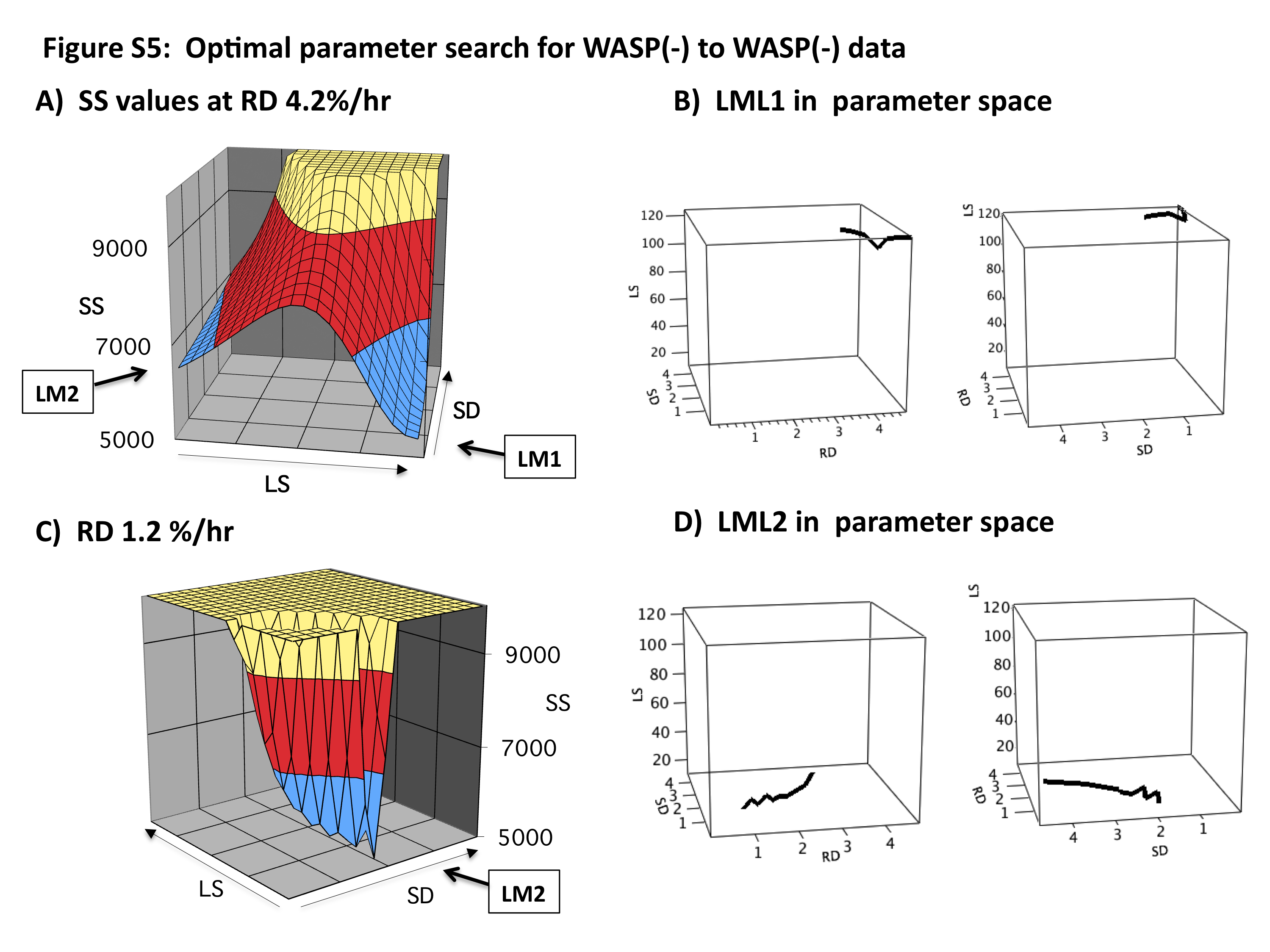

Supplement: Figure S5 — Optimal parameter search for WASP(-) to WASP(-) data. A) SS values were determined for each of 400 points in a plane in equilibration-verified parameter space defined by RD = 4.2%/hr. The LS axis range is 10 to 124 hr (resolution 6 hr)(left to right). The SD axis range is 0.01 to 4.751 (resolution 0.25). The equilibration phase duration is 500 hr. B) Three dimensional plot of the LM1 SS minima identified in 20 consecutive RD-defined planes of parameter space. The plot on the right is equivalent to that on the left, but rotated 90 degrees on the Z-axis. The LM could not be identified below an RD of 3.4%/hr. C) The same process was used to define SS values in a plane defined by RD = 1.2%/hr. The LS axis range is 10–124 hr. The SD range is 0.01 to 4.751 (resolution 0.25). LM2 in this plane is defined by SD = 1.75 and LS = 16 hr. The Equilibration phase is 500 hr. D) Three dimensional plot of the LM2 SS minima identified in 20 consecutive RD-defined planes of parameter space. The plot on the right is equivalent to that on the left, but rotated 90 degrees on the Z-axis. (TIFF) [file pone.0055087.s005.tif]

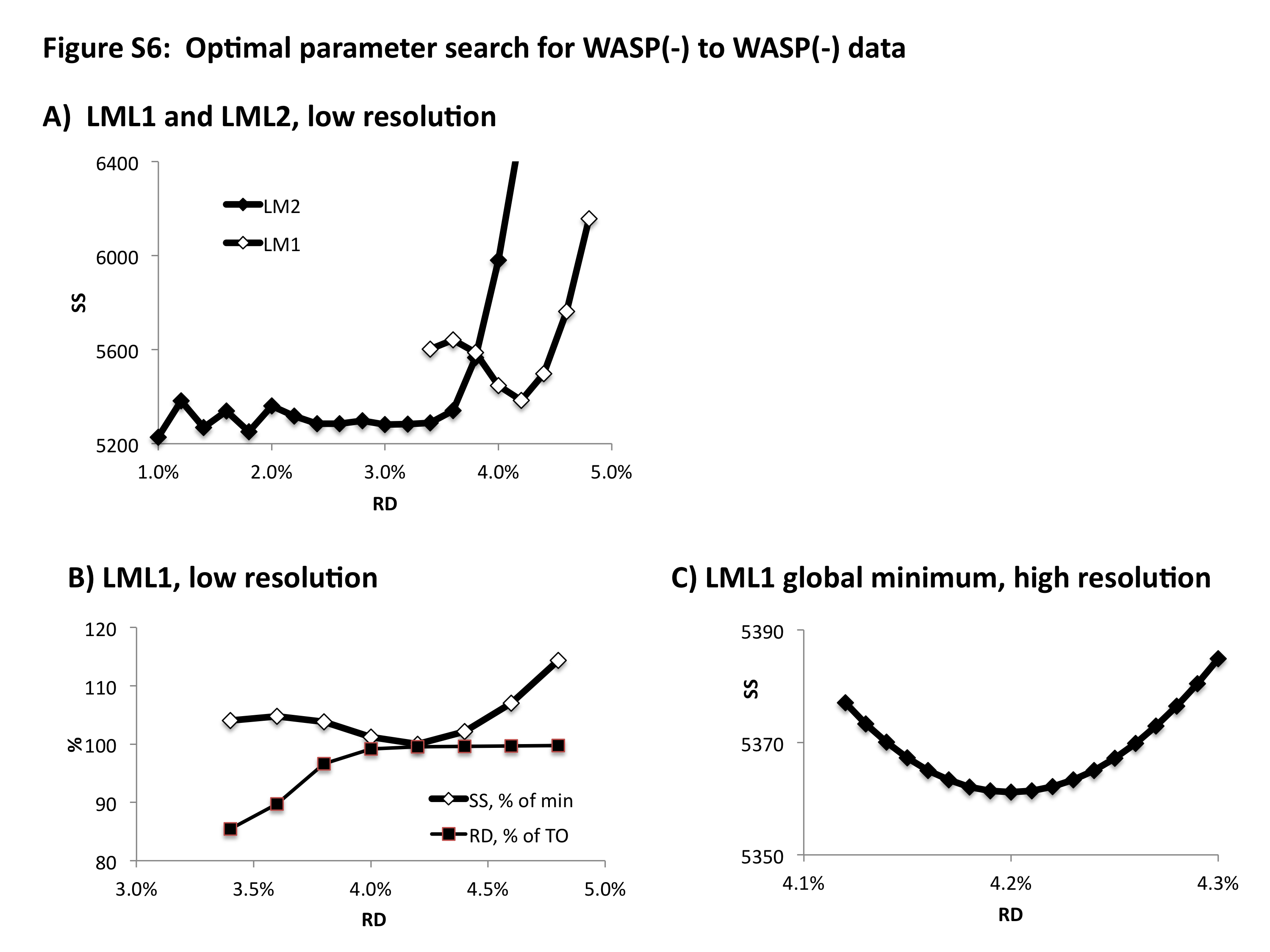

Supplement: Figure S6 — Optimal parameter search for WASP(-) to WASP(-) data. A) SS values are shown for the LML’s described in the previous figure. B) Diamonds: SS values for LML1 are shown, normalized to their minimum. Squares: RD as % of total platelet turnover is shown for the same LM points. At the global minimum identified in this study (RD = 4.2%/hr), RD accounts for over 99% of platelet turnover. C) High resolution study of LM1. The equilibration-verified parameter ranges were RD 4.12–4.13% (resolution 0.01%), LS 110–148 hr (resolution 2 hr), SD 0.01–0.3 (resolution 0.15). The global minimum identified in this study is shown in table 1. (TIFF) [file pone.0055087.s006.tif]

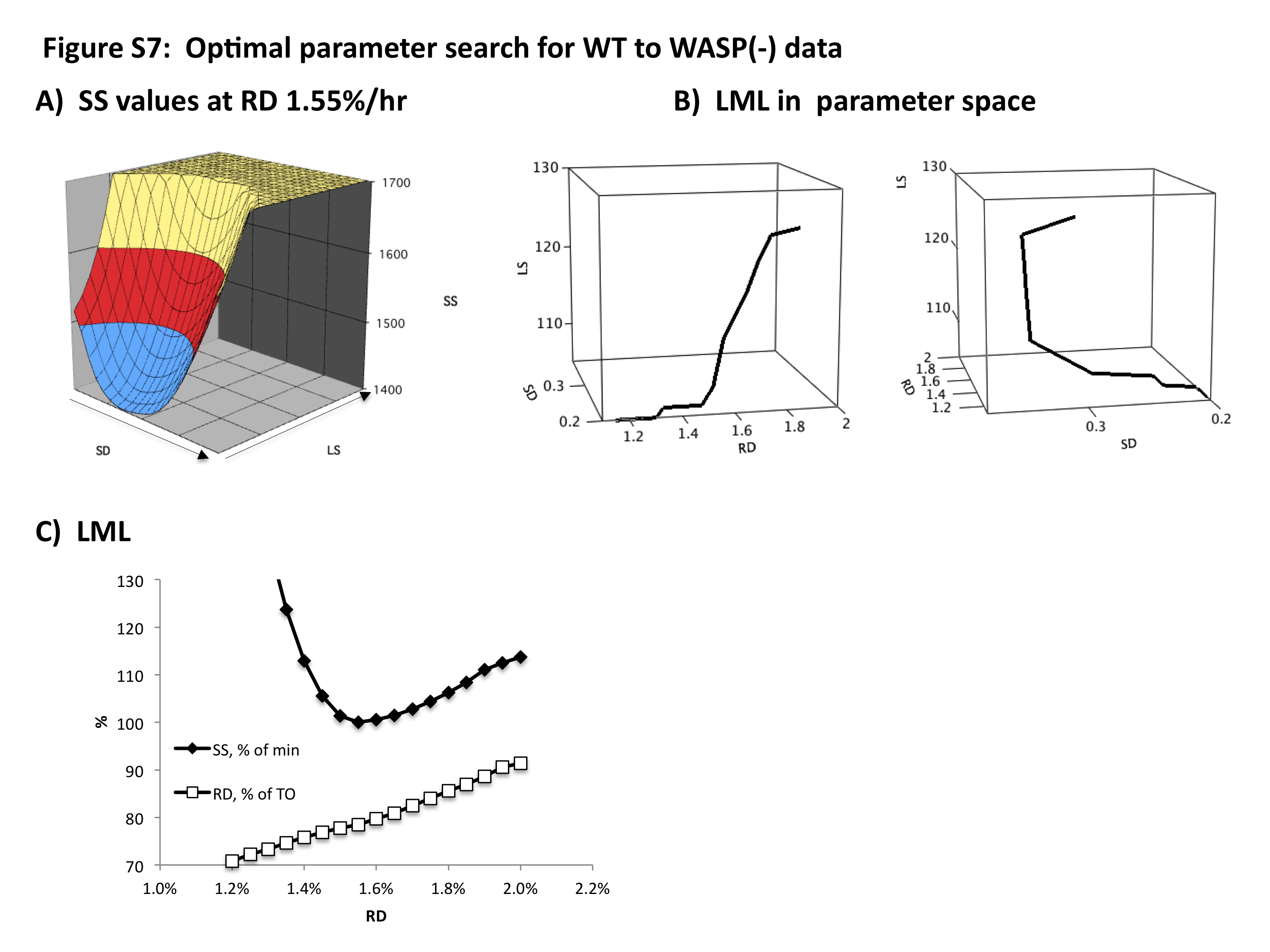

Supplement: Figure S7 — Optimal parameter search for WT to WASP(-) data. A) SS values were determined at the RD value shown. Ranges evaluated for SD were 0.1–0.575 (resolution 0.025; for LS, 105–124 hr left to right (resolution 1 hr). B) LM1 was evaluated in 20 consecutive RD-defined planes (range 1.1–2.1%/hr, resolution 0.05%/hr) over the LS and SD ranges described in A. All Local minima at RD values of 1.6% or less occur at an LS of 105 hr. C) Diamonds: SS values for the LML shown in (B), normalized to the global minimum. Squares: RD as % of turnover rate for the same LM points. (TIFF) [file pone.0055087.s007.tif]

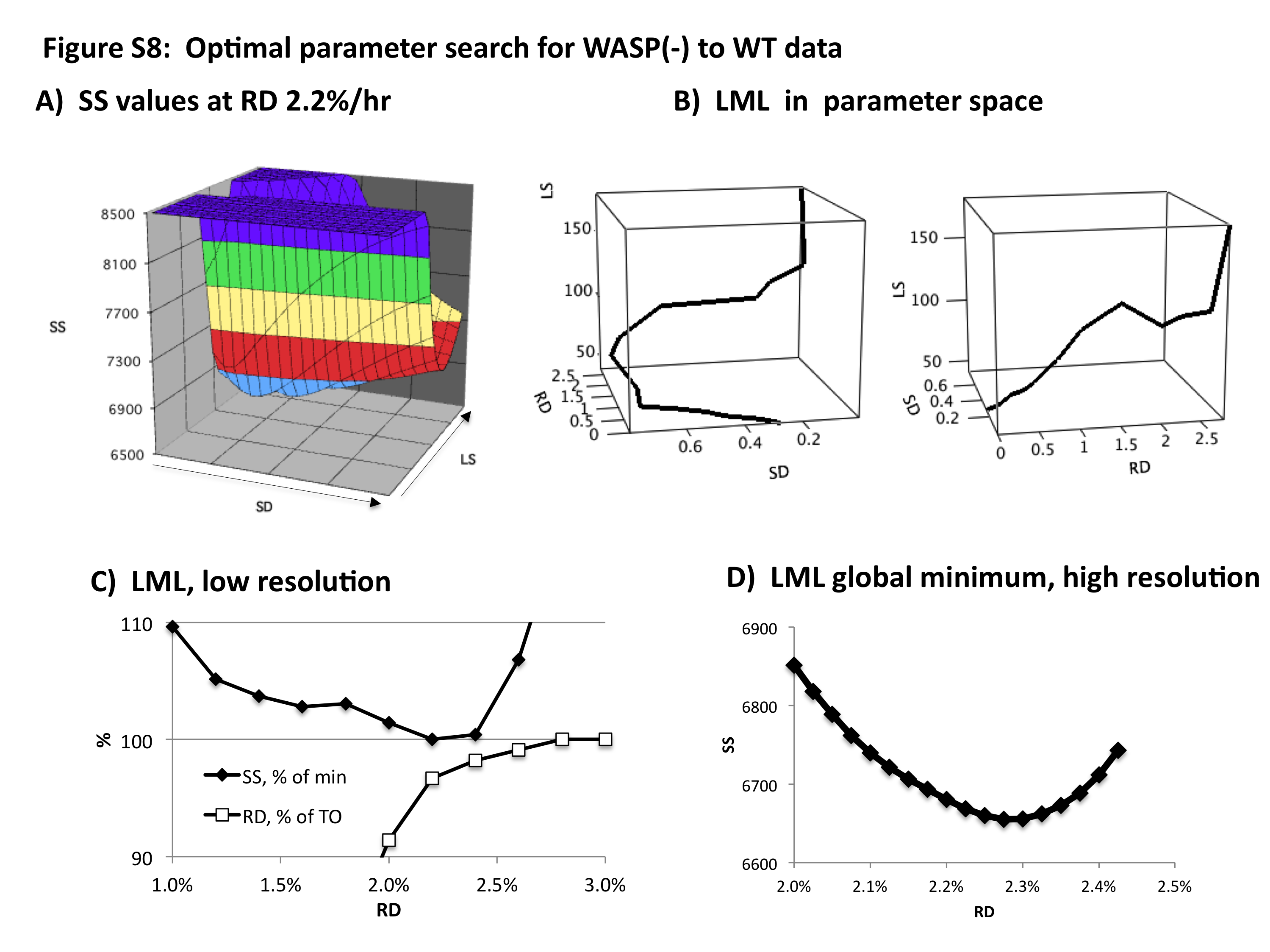

Supplement: Figure S8 — Optimal parameter search WASP(-) to WT data. A) SS values were determined in the RD plane shown. Ranges evaluated for SD were 0.001–0.92 (resolution 0.03); for LS, 1–191 hr (resolution 10 hr). B) LML was evaluated in 16 consecutive RD-defined planes. Range: 0–3.8%/hr (resolution 0.2%/hr). The second 3D plot shows the LML rotated 90 degrees on the vertical axis. C) SS values for the LML shown in (B) are expressed as a percentage of their minimum value (Black diamonds). RD values for the same LML are expressed as a percentage of the total platelet turnover rate. D) A high resolution study of the global minimum in (C). Ranges evaluated are RD 1.95–2.45, LS 96–115, and SD 0.1–0.4. Resolution is 5% of each range. The resultant global minimum is shown in table 1. (TIFF) [file pone.0055087.s008.tif]
