# Supplementary material for: A Numerical Analysis Model for the Interpretation of In Vivo Platelet Consumption Data
Source: PLoS One. 2013 Jan 28;8(1):e55087. doi: 10.1371/journal.pone.0055087 (PMC3557263; doi:10.1371/journal.pone.0055087)
Supplement: Text S1 — Additional details on optimal parameter searches and other statistical methods. (DOCX) [file pone.0055087.s012.docx]

**Optimal parameter search method:**

*Parameter range validation.*  In order to define the range of parameter values in which the model achieves equilibration, we evaluated the behavior of (e) (defined in the text) over a range of parameter value sets. **Figure S1** shows that (e) consistently declines with increases in LS and SD, and declines as RD is reduced. Based on these observations, we generated contour maps of (e) values in a series of planes in parameter space defined by SD values. Such a contour map (**figure S2**) allows us to visualize a rectangular range of RD and LS values in which equilibration is adequate. Comparison of this map to a similar map at a lower SD value (**figure S3**) allows us to define a cuboidal volume of parameter space within which all points generate an adequately equilibrated platelet population. Definition of such a volume is required for optimal parameter searches. To minimize computation time, we have at different points in this study defined these volumes for two different models, one with a 240 hour equilibration phase and one with a longer (500 hour) equilibration phase. These models entail matrix sizes of (240 x 365 = 87,600) and (500 x 625 = 312,500), well within the technical limitations of the software. PS volumes in which equilibration has been verified are referred to as equilibration-verified volumes.

*Consumption of platelets from syngeneic donors:*  To maximize the range of parameter space evaluated for the WT to WT platelet consumption data set, it was necessary to evaluate the three overlapping, equilibration verified PS volumes shown in **table S1**. Definition of these three volumes was needed to ensure model equilibration, as a single cuboidal PS volume encompassing all three contains points for which the model does not equilibrate adequately. Local minima (LM) in each PS volume were identified as shown in **figure 3A**. The local minimum line (LML) we identified in these three volumes is shown in 3D plots of PS in **figure S4a**. SS values for each point on the LML are shown in **figure S4b**. Those values (as a percentage of the global minimum value) are plotted with the associated RD values as % of the net turnover rate. Lack of intersection of the two curves demonstrates the validity of estimating both lifespan-dependent and random consumption from this data. A subsequent high resolution study (**figure S4c**) resolved the global minimum at the parameter values shown in **table 1**. The associated consumption curve, plotted against the data used to identify it, is shown in **figure 4**.

Optimal parameter searches for data obtained with WASP(-) platelets in WASP(-) recipients yielded two local minimum lines in parameter space: LML1 at high RD and LS values, and LML2 at low RD and LS values and high SD values. Examples of the local minima are shown in **figure S5(a and c).** LML1 and LML2 are shown in 3D plots of parameter space in **figure S6(b and d)**. Plots of the SS values associated with these LML’s are in **figure S6(A)**.

The trajectory of LML1 toward high RD, high LS, and low SD values corresponds with minimization of lifespan-dependent consumption. This is evident at the global minimum of LML1 (**figure S6b)**, a point at which RD comprises over 99% of the population turnover rate. A high resolution study of LML1 is shown in **figure S6c**. Evaluation in a PS including a higher LS range extends LML1 toward parameter values showing an even lower fraction of lifespan-dependent turnover, and an indistinguishable global minimum RD value (data not shown). These features correspond to a purely exponential (random) consumption process for which optimal LS and SD values are not evaluable. Linear regression analysis of a semi-log plot of consumption vs. time (**figure S9**) corroborates this conclusion.

LML2 for the WASP(-) to WASP(-) data set is a degenerate minimum line (it shows a similar SS value over a wide range of RD values on the LML, see **figure S6a**). Detection of LML2 is a consequence of the fact that at as SD values approach low LS values, the “lognormal” consumption curve shifts from a sigmoidal appearance to one that is almost indistinguishable from simple exponential consumption.

**Resampling method:** To estimate standard deviations for parameter values, we performed the same type of analysis on data subsets generated by “jacknife” resampling. This entailed analysis of n data subsets derived from each of the data sets shown in **table 1,** where n is the number of recipient mice studied in each data set. Because this required a large number of calculations, we evaluated a smaller equilibration-verified parameter space volume (10 x 10 x 10) for each data subset, with a 240 hour equilibration phase. The volumes evaluated for each subset are shown in **table S2**. This process allowed us to identify a standard error value for each of the optimum (global minimum) parameter values shown in **table 1,** with the exception of the LS value for the WT to WASP(-) data (discussed below).

**Consumption of platelets from allogeneic donors:** To evaluate the consumption of WT platelets in WASP(-) recipients, the relevant donor values (platelet cohort populations and cohort CRD values at i=240 hr; platelet production rate) were grafted into row 240 of the same model used to generate the donor data. (Note that equilibration of the donor populations was verified within the PS volume used to identify the optimal parameter values in autologous recipients, above). We note that the model cannot quantify any potential recipient-dependent decrease in the LS of the donor platelets. Such quantification would require evaluation of at least one additional parameter (specifically, a determinant of the rate of clearance of those donor platelets which have exceeded, at the time they are injected, a hypothetical recipient-dependent LS). The model is sensitive, however, to any recipient-dependent increase in LS. We therefore evaluated optimal values in a parameter space defined by LS values of 105 hr or greater. The resultant LML for the WT to WASP(-) data set is shown in **Figure S7(B)**; the global minimum is identified in **figures S7(A);** at higher resolution in **figure S7(C);** and is shown in **table 1.** The results show no recipient-dependent increase in the LS of WT platelets.

To evaluate the consumption of WASP(-) platelets in WT recipients, a similar process yielded the LML shown in **figure S8b**. Unlike the case for the WT to WASP(-) analysis, this LML reached a global minimum at a point in PS at which LS-dependent consumption still contributed significantly to the overall turnover rate (**figure S8c**) inasmuch as it allowed estimation of a non-zero optimal value of RD (**table 1**). Optimal parameter values were subsequently resolved at high resolution (**figure S8d**).
